# Die Einführung der Antipsychotika an der Neurologisch-Psychiatrischen Klinik der Universität Leipzig und ihre Auswirkungen auf andere Therapieformen sowie auf die Verweildauern und Verlegungen

**DOI:** 10.1007/s00115-020-00931-y

**Published:** 2020-06-15

**Authors:** Christian Oeser, Holger Steinberg

**Affiliations:** 1 Forschungsstelle für die Geschichte der Psychiatrie, Klinik und Poliklinik für Psychiatrie und Psychotherapie, Medizinische Fakultät der Universität Leipzig, Semmelweisstr. 10, 04103 Leipzig, Deutschland; 2 Klinik für Psychiatrie und Psychotherapie im Fachkrankenhaus Hubertusburg, Wermsdorf, Deutschland

**Keywords:** Geschichte der Psychiatrie, 20. Jahrhundert, Deutschland, Therapie, Psychiatrische Versorgung, History of psychiatry, 20th century, Germany, Therapy, Psychiatric care

## Abstract

**Ziel:**

Die Auswirkungen der Einführung der Antipsychotika auf die psychiatrische Versorgung in einer Klinik sollen erstmals überhaupt untersucht werden – konkret in der Neurologisch-Psychiatrischen Klinik der Universität Leipzig von 1946 bis 1965.

**Forschungsfragen:**

Zu welchem Zeitpunkt wurden welche Antipsychotika erstmals eingesetzt, in welchem Umfang und wie wirkte sich dies auf den Einsatz der traditionellen Therapieformen aus.

**Material und Methode:**

Nach psychopathologischen Kriterien wurden 306 Fälle aus dem Bestand an Patientenakten aus dem Archiv der Klinik ausgewählt und anhand der Fragestellungen systematisch erfasst.

**Ergebnisse:**

Der Einsatz der Antipsychotika begann ab 1953 mit zunehmender Häufigkeit und Dauer. Traditionelle Therapien und Antipsychotika wurden häufig in Kombination eingesetzt. Insbesondere der Einsatz von Antipsychotika und Elektrokrampftherapie stellte die neue Basistherapie dar. Eine Herabsenkung der Verweildauer konnte nicht nachgewiesen werden. Ab 1955 erfolgten jedoch weniger Verlegungen in die Landesheilanstalten und mehr Patienten konnten als „gebessert“ in die Häuslichkeit entlassen werden. Ab 1961 konnten erste Vermerke zu Entlassungsmedikationen gefunden werden und es fanden sich Hinweise auf ambulant durchgeführte Behandlungen wie z. B. auch Krampftherapien.

**Diskussion:**

Die Erleichterung der psychischen Rehabilitation durch die Antipsychotika kann nicht direkt belegt werden, jedoch liegt es nahe, dass die Gabe über eine zeitlich begrenzte „Kur“ hinaus zu dieser Entwicklung und auch zur Etablierung ambulanter Strukturen beigetragen haben. Somit konnte sowohl ein Einschnitt in der psychiatrischen Behandlung als auch ein Wandel für die Patienten selbst ausgemacht werden.

## Abstract (en)

**Aim:**

The impacts of the introduction of antipsychotics on psychiatric care in a clinic were investigated for the first time, specifically in the Neurological and Psychiatric Clinic of the University of Leipzig from 1946 to 1965.

**Research questions:**

When and which antipsychotics were first used, to what extent and how did this affect the use of traditional forms of therapy?

**Material and methods:**

According to psychopathological criteria, 306 cases were selected from the database of patient records from the hospital archive and systematically reviewed with respect to the research question.

**Results:**

The use of antipsychotics began in 1953 and subsequently with increasing frequency and duration. Traditional therapies and antipsychotics were frequently used in combination. In particular, the use of antipsychotics and electroconvulsive therapy represented the new basic therapy. A decrease in the length of hospitalization could not be demonstrated. From 1955, however, fewer transfers to the provincial hospitals were necessary and more patients could be discharged into domestic life as “improved”. Beginning in 1961, for the first time a discharge medication was mentioned and evidence of outpatient therapies including electric shock therapies were recorded.

**Conclusion:**

Evidence of facilitation of mental rehabilitation through the use of antipsychotics cannot be directly confirmed; however, it appears that the administration beyond a time-limited treatment has contributed to this development and also to the establishment of outpatient facilities. Thus, both a watershed in the psychiatric treatment and a change for the patients themselves could be identified.

## Hintergrund

In der Geschichte der Psychiatrie gelang es ab Mitte der 1950er-Jahre, mit der Einführung der modernen Psychopharmaka die bisherigen Behandlungsarten psychiatrischer Erkrankungen ganz wesentlich zu ergänzen. Für diesen Wandel hat sich sogar der Terminus der „Revolution der psychiatrischen Therapie und Versorgung“ etabliert. Neben der Behandlung depressiver Störungen mithilfe der ersten Antidepressiva sollte insbesondere die der Schizophrenien und Manien mit der Einführung der ersten Antipsychotika einen deutlichen therapeutischen Wandel erfahren. Jean Delay, der seit 1942 als Leiter der Pariser Saint-Anne-Klinik wirkte, fand in dem Medikament Chlorpromazin eine Alternative zum bisherigen „lytischen Cocktail“ ohne die Komplikation einer Abhängigkeitsentwicklung, wie sie bei den zuvor angewandten Barbituraten zu verzeichnen gewesen war. Er und sein Assistent Pierre G. Deniker stellten unter längerfristiger Chlorpromazinbehandlung Behandlungserfolge bei schizophrenen Psychosen fest, vor allem eine Dämpfung von Wahngedanken, eine Minderung der Aggressivität und eine beruhigende Wirkung auf das Verhalten. Sie berichteten der Fachwelt über ihre Behandlungserfolge bei „schweren“ und sogar „refraktären“ Fällen auf Kongressen und in Publikationen ab 1952 [7]. Ihre Ergebnisse wurden schnell auch durch weitere Untersuchungen bestätigt [37]. Auch über den Einsatz des Chlorpromazins als schnelles Mittel gegen psychomotorische Erregungszustände wurde wiederholt berichtet [9]. Bis zur Einführung der Antipsychotika hatten „Schockbehandlungen“ mittels Insulin, Cardiazol und v. a. Elektrokonvulsion sowie die Behandlung mittels sedierender Medikamente dominiert. Weiterhin waren konservative Therapien wie Psychotherapie oder Arbeitstherapie, aber auch Badetherapien eingesetzt worden [8].

Obgleich Arzneistoffe und deren Verabreichung und Wirkung seit ca. 15 Jahren ein zentrales Thema der Medizingeschichtsforschung [3] sind, liegen erstaunlicherweise über die konkrete Einführung der ersten modernen Psychopharmaka in die Kliniken und deren Auswirkungen auf das angewandte Spektrum von Therapien bis heute kaum Forschungsarbeiten vor. Dies trifft noch stärker auf die Ostblockstaaten und die DDR zu [12]. Balz leistete entsprechende Vorarbeiten in ihrer Forschung zu den Bemühungen, eine Standardisierung der klinischen Wirksamkeit der ersten Antipsychotika in der Bundesrepublik zu erreichen. So berichtete sie vor allem über die Einführung des Chlorpromazins an der Heidelberger Psychiatrischen Universitätsklinik – auch über den ersten Patienten, der überhaupt mit Megaphen behandelt wurde [1, 2]. Zur Psychiatrie in der DDR legten Balz und Kollegen eine Arbeit über die zentralistische Planung und Kontrolle der Forschung und Entwicklung der Psychopharmaka in den 1960er-Jahren vor [14] und gingen der Frage nach, inwiefern an der Psychiatrischen Klinik der Charité Überwachungsroutinen durch den Einsatz von Psychopharmaka, vor allem Antidepressiva und Beruhigungsmitteln, verändert wurden [4]. Wie häufig konkret die neuen Medikamente verordnet wurden und ob bzw. welchen Einfluss das auf die anderen verfügbaren Therapien hatte, lag nicht in ihrem oder dem Forschungsfokus anderer. Jedoch entwickelten Balz und Kollegen die interessante These, dass parteilinientreue Psychiater, die nicht an gesellschaftlichen Tabuthemen rührten und mit der Propagierung der Psychopharmaka eine kostengünstigere Behandlungsmethode verhießen, sozialpsychiatrisch-reformorientierte Ansätze der 1950er- und 1960er-Jahre verdrängten [5].

Im Rahmen dieser Studie soll erstmals konkret anhand einer größeren Anzahl von Patientenakten über den Wandel der Therapieformen bei Schizophrenien und Manien vor dem Hintergrund der Einführung der Antipsychotika berichtet werden. Ausgewählt wurde dazu die Neurologisch-Psychiatrische Klinik der Universität Leipzig in der ehemaligen DDR.

Der Einführung der Antipsychotika war eine Überarbeitung der Lehrmeinung über die Genese der Schizophrenie vorausgegangen. Das von Gottfried Ewald verfasste Lehrbuch der Psychiatrie von 1948 [8] widmete der Schizophrenie detaillierte Krankheitsschilderungen und Überlegungen zur Pathogenese. Insbesondere wies Ewald nach, dass diese Krankheitsbilder nicht das Ergebnis einer endokrinen Störung von Hoden bzw. Ovar sowie anderer innersekretorischer Organe sein könnten, auch Stoffwechselstörungen ordnete er als allenfalls vereinzelte Ursache ein. Da sämtliche zur Verfügung stehenden diagnostischen Methoden keine verlässlichen Resultate erbracht hätten und da auch histologische Untersuchungen vermeintlich betroffener Areale keine Pathologien hätten darstellen können, schlussfolgerte Ewald, dass Schizophrenien und Manien am ehesten das Ergebnis einer Funktionsstörung des Gehirns seien. Aus diesem Grund solle die Akutbehandlung vorerst symptomatisch und mit dem Ziel erfolgen, weitere Behandlungen überhaupt erst einmal zu ermöglichen. Für diese Akutbehandlung wurden Scopolamin sowie Morphininjektionen zur Beruhigung empfohlen. Es könnten auch Dauerbäder in körperwarmen Wasser, bei stark erregten Patienten notfalls durch das Einbinden in Leinen verstärkt, oder eine Dauerschlaftherapie zur Anwendung kommen, bevor nach erfolgreicher Ruhigstellung zur Arbeitstherapie übergegangen werden könne. Die Arbeitstherapie sei insbesondere deshalb zu einem „Hauptfaktor der Therapie“ geworden, um die Kranken nicht „im Bett versacken“ zu lassen. Neben den beschriebenen Methoden fanden auch die „somatisch-ärztlichen Kuren“ Erwähnung, womit vor allem die Insulinkrampftherapie, aber auch die Cardiazol- und Elektrokonvulsionstherapie gemeint waren. Insbesondere die teilweise mehr als drei Monate anzuwendende Insulintherapie wurde als Erfolg verheißend beschrieben.

Die Elektrokonvulsionstherapie hatte sich, vermutlich da sie leicht anzuwenden war, während der 1940er-Jahre als Standardtherapie vor allem bei akuten Zuständen und schweren wie auch katatonen Formen der Schizophrenien durchgesetzt. Im vielerorts kriegszerstörten Deutschland konnten die Kliniken zumeist auf entsprechende Geräte aus der Zeit vor dem Krieg zurückgreifen [31]. Die Nebenwirkungen der Elektrokonvulsionstherapie wurden unterschiedlich interpretiert. In der Kaufbeurener Heil- und Pflegeanstalt wurde der zuweilen zu beobachtende Verlust des Gedächtnisses teilweise auch als positiv angesehen, da dadurch eine Wiederholung der Behandlungen vereinfacht werde [26]. Gelegentlich wurden als Nebenwirkungen auch Knochenbrüche und Luxationen beobachtet. Jedoch gab es weitere und schwerwiegendere Nebenwirkungen wie Blutdruckschwankungen, Zyanosen, Temperaturanomalien, Blutbildveränderungen, Veränderungen der Blutsenkungsgeschwindigkeit und des Blutzuckers sowie der Blutgerinnung [36]. Die Erfolge der Elektrokonvulsionstherapie (EKT) untersuchte Walter Schulte an der Universitäts-Nervenklinik in Jena. Nach seinen Untersuchungen erreichte die EKT bei Schizophrenien und Manien immerhin bei ca. 10 % eine bleibende deutliche Besserung sowie bei weiteren 40 % eine bleibende, jedoch nur geringe Besserung der vorhandenen Symptomatik. Bei der Hälfte der Fälle jedoch war gar keine Wirkung zu verzeichnen. [28].

Dieser physikalischen Behandlungsform stand die mittels medikamentöser Agenzien gegenüber. Im Jahr 1923 entwickelte Karl Friedrich Schmidt in Finnland Pentamethylentetrazol, welches ab 1924 von der Firma Knoll [15] auf den Markt gebracht wurde. Dieses wurde intravenös mit dem Ziel verabreicht, einen tonisch-klonischen Krampfanfall auszulösen. Die Patienten litten bei dieser Therapie allerdings oft unter einer „quälenden Aura“ [16, 24] oder gar „Vernichtungsangst“ [37]. Ladislas J. Meduna, der das Pentetrazol 1934 als krampfauslösendes Mittel auch bei Schizophrenien einführte, sah eine Erfolgsquote der Cardiazolkrampfbehandlung zwischen 30,2 und 53,5 % [17]. Mitte der 1930er-Jahre schlug Manfred Sakel eine weitere Therapieoption vor, bei welcher durch Insulinverabreichung eine Hypoglykämie und ein mehrere Minuten anhaltendes Koma und sowie ein Krampfanfall herbeigeführt wurden [25]. Als Nebenwirkungen der Insulintherapie beschrieb Richard Heidrich aufgrund seiner Analyse der an der Charité vorgenommenen Behandlungen mit Insulin unter anderem Hemiplegien durch zerebrale Blutungen, Dysarthrien, Aphasien, infantiles Sprechen, Parkinsonismus, Hirnnervenlähmungen, Status epilepticus, Korsakow-Syndrome, Dämmerzustände und Stupore, Zeitrafferphänomene, anaphylaktische Schocks, Pneumonien und Lungenödeme [10].

Ab den 1950er-Jahren kann man zunehmend von einem neurobiologischen Forschungsansatz sprechen [11]. Bereits in den Jahren 1953 und 1954 entstanden Hypothesen über vermutete und zunehmend gesicherte pharmakologische Mechanismen hinsichtlich der intrazerebralen Signalübertragung. So wurde neben dem bekannten Adrenalin und Noradrenalin von Betty Twarog und Irvine Page das Serotonin entdeckt [35, 38]. Bereits 1955 wurde dieser Botenstoff in seiner Wirkung mit der bereits aus Tierversuchen bekannten Wirkung von Reserpin verglichen. Außerdem wurde mit Dopamin eine weitere entscheidende Transmittersubstanz entdeckt. In der Folge wurde die Hypothese formuliert, dass Schizophrenien ursächlich durch eine endogene Erhöhung des Dopaminspiegels sowie eine gesteigerte Sensitivität der Dopaminrezeptoren bedingt seien [6]. Somit wurde erstmals ein genauer Mechanismus für ein funktionelles Versagen ermittelt, das bereits Ewald in seinem 1948 erschienenen Lehrbuch postuliert hatte, nachdem alle Versuche, ein organisches Substrat nachzuweisen, gescheitert waren [8]. Dieses Postulat, dass Schizophrenien auf einer Funktionsstörung des Gehirns beruhten, war es auch, das die Aufmerksamkeit in Richtung einer „stoffgebundenen“ somatischen Therapie schizophrener Erkrankungen lenkte. Als ein mögliches Präparat wurde 1951 von der Firma Rhône-Poulenc Chlorpromazin entwickelt und im April 1951 an die im Hôpital Paul-Brousse in Villejuif tätigen Psychiater Jean Sigwald und Daniel Bouttier verschickt, welche nach Anwendung bei einer Patientin eine Veränderung ihres Wesens, eine Normalisierung ihres Schlafes sowie eine deutliche Reduktion der Beeinträchtigung ihres Allgemeinzustandes durch Halluzinationen und Verfolgungsideen beobachteten [29]. Des Weiteren wurde nach einem Selbstversuch des Schweizer Chemikers Albert Hofmann die halluzinogene Wirkung von LSD als „experimentelle Schizophrenie“ beschrieben [20] und gleichzeitig in eben diesem Selbstversuch nachgewiesen, dass die Verabreichung von Chlorpromazin diese beenden könne.

Die größer angelegten Studien an 300 Patienten von Jean Delay und Pierre G. Deniker folgten. Beide wiesen auf erste vielversprechende Ergebnisse auf der Hundertjahrfeier der Societé Médico-Psychologique 1952 hin. Die bekannt gewordenen Therapieerfolge bei paranoiden Schizophrenien wurden von der deutschen Psychiatrie im Vergleich zu denjenigen bei Elektroheilbehandlung und Insulinkrampftherapie schnell als wirksamer anerkannt und im Gegensatz zu denjenigen bei den Defektbildern und bei Hebephrenien als nachhaltiger gewürdigt [18, 22].

In der BRD wurde 1953 durch die Firma Bayer das erste Phenothiazinpräparat Megaphen als Neuroleptikum auf den Markt gebracht [23]. Zeitnah wurde dieses auch in die DDR importiert. Es ist jedoch überliefert, dass Megaphen keineswegs unbegrenzt in der DDR eingesetzt werden konnte, da entsprechende Devisen rar waren [31]. Mit der Einführung der Politik der „Störfreimachung“ ab 1961, die das Ziel verfolgte, die DDR von der BRD vollkommen unabhängig zu machen, nahmen derartige Limitationen noch zu [4]. Dem Hydrierwerk in Rodleben war es freilich gelungen, das Megaphen sehr schnell für die DDR nachzubauen – als international nicht anerkannter Staat brauchte die DDR auch keine Sanktionen für diese Patentverletzung zu fürchten. So wurde Megaphen bereits 1954 unter dem Namen Propaphenin in der DDR zugelassen und die Regelproduktion begonnen [13]. Ab 1955 wurde das Originalpräparat nur noch für die Narkoseeinleitung weiter importiert [19]. Was die Wirksamkeit anging, hielt Propaphenin jedem Vergleich mit dem Originalmedikament stand. Allerdings war auch die Verfügbarkeit des Nachahmungspräparats gerade in der Anfangszeit noch stark begrenzt [9]. In einer Studie über Erfahrungen mit der Anwendung von Megaphen wies Dietfried Müller-Hegemann, 1952 bis 1964 Direktor der Leipziger Neurologisch-Psychiatrischen Universitätsklinik, darauf hin, dass das Antihistaminikum Thiantan, welches auch in der DDR produziert wurde, eine ähnliche Wirkung habe wie Megaphen [21]. Anfänglich erfolgte der Einsatz der Antipsychotika in Form zeitlich limitierter Kuren von wenigen Wochen, was sich jedoch änderte, als deutlich wurde, dass die Behandlung nicht kausal erfolgte, sondern syndromorientiert. In seinem Lehrbuch von 1966 empfahl Müller-Hegemann den Einsatz einer Kombination von Antipsychotikakur mit zehn Anwendungen der Elektrokonvulsionstherapien, bei ausbleibendem Therapieerfolg sollten unter dieser Kombination zudem zusätzlich Insulinkomaanwendungen zum Einsatz kommen. Im Jahr 1961 wurde in den USA eine Multicenterstudie des National Institute of Mental Health an 9 Kliniken durchgeführt, die die hohe Effizienz der Chlorpromazintherapie bestätigte. Jedoch wurde bezüglich des Wirkmechanismus eine große Spannbreite von Erklärungen vertreten. Tiefenpsychologisch orientierte Ärzte vermuteten einen reinen Placeboeffekt, andere Ärzte diskutierten den durch die Medikation ermöglichten, bereits breiten Einsatz sozialpsychiatrischer Behandlungselemente als den hauptsächlichen Effektträger [37]. Die Lehrmeinung wandelte sich durch die Erfahrungen nach der Einführung der Antipsychotika. Das von Rudolf Lemke begründete Standardlehrbuch der DDR für die Neurologie und Psychiatrie spiegelte in seiner 3. Auflage von 1965, die von Helmut Rennert bearbeitet worden war, die therapeutischen Neuerungen wider. So stellte der Hallenser Lehrstuhlinhaber die Behandlung mittels traditioneller Methoden wie Elektrokrampfbehandlung, medikamentöser Krampfbehandlung durch Insulin und Cardiazol und die Leukotomie zwar noch vor, jedoch betonte er, dass Cardiazolkrampfherapien und Leukotomien nicht mehr angewendet würden. Vor allem aber arbeitete er heraus, dass sich durch die Einführung der Antipsychotika das Klima, gerade auch auf den geschlossenen Stationen, zu einem „Heilklima“ entwickelt bzw. gewandelt habe [16]. Müller-Hegemann sprach in seinem Lehrbuch von 1966 [22] bereits von einem „Siegeszug der Neuroleptika“, welche die therapeutischen Erfolge bei der Behandlung der Schizophrenien bedeutend vergrößert hätten. Dies habe die durchschnittlichen Aufenthaltsdauern der Patienten beträchtlich verkürzt, teilweise sogar zu einer regelrechten „Entleerung der psychiatrischen Spitäler“ geführt. Müller-Hegemann verweist aber auch auf die Nebenwirkungen und mahnt zur Zurückhaltung bei der Medikamentengabe. In akuten Stadien sei eine Phenothiazintherapie mit der Elektrokonvulsionstherapie zu kombinieren. Im Falle einer Therapieresistenz sei eine intensive Insulinkrampftherapie indiziert. Nach Beendigung der Akuttherapie empfahl Müller-Hegemann eine Erhaltungstherapie mittels Propaphenin.

## Methodik

Für die retrospektive Auswertung wurden 400 Patientenakten der Neurologisch-Psychiatrischen Universitätsklinik Leipzig mit der Diagnose Schizophrenie und 100 Akten mit der Diagnose Manie aus den Jahren 1946 bis 1965 mit der Absicht erfasst, einen Einblick in die jeweils verordneten Behandlungsmethoden und deren möglichen Wandel vor, während und nach der Einführung der Antipsychotika zu erhalten. Diese Akten sind im Bestand der Patientenakten der Neurologisch-Psychiatrischen Universitätsklinik Leipzig im Universitätsarchiv archiviert [39]. Die Eruierung der einzelnen Akten erfolgte auf der Grundlage der für jede Akte existierenden Dateikarte, die nach den Eingangsdiagnosen geordnet sind. Kriterien für die Aufnahme in die Studie waren einerseits das Vorliegen psychopathologischer Symptome einer Schizophrenie (Wahnerleben und Halluzinationen, desorganisierte Sprache, desorganisiertes Verhalten, Gedankenmanipulation, katatone Symptome oder Negativsymptomatik wie z. B. Affektverflachung, Sprachverarmung oder Apathie) bzw. andererseits einer Manie nach Aufnahmeanamnese. Nicht einbezogen wurden Fälle, bei denen die schizophrenen oder manischen Symptome als mit hirnorganischen Erkrankungen, Depressionen oder Intelligenzminderungen zusammenhängend beschrieben wurden. Ebenso waren stationäre Aufnahmen unter exogenem Substanzeinfluss, Patienten, bei denen hirnchirurgische Eingriffe wie Lobotomien vorgenommen wurden, und Aufnahmen für reine Begutachtungen nicht Gegenstand der Betrachtung. Übrig blieben 306 Fälle mit der Diagnose „Schizophrenie“ (262) oder „Manie“ (44). Tab. 1 gibt einen Überblick über die in diese Studie inkludierten Fälle nach dem Jahr der Aufnahme.

**Table 1:** 

Jahr	Weiblich	Männlich	Gesamt	Alter		
				Minimum	Maximum	Mittelwert
1946	10	9	19	16	49	22
1947	17	7	24	16	70	33
1948	16	3	19	18	69	38
1949	19	2	21	16	51	32
1950	13	5	18	17	55	28
1951	10	7	17	24	53	38
1952	10	8	18	18	55	32
1953	9	11	20	17	56	35
1954	12	7	19	16	59	37
1955	10	10	20	18	58	32
1956	9	25	34	11	62	38
1957	6	13	19	17	53	33
1958	11	4	15	19	63	33
1959	7	11	18	16	70	38
1960	5	4	9	27	65	39
1961	1	7	8	17	66	45
1962	2	1	3	36	67	48
1963	0	4	4	30	0	40
1964	0	0	0	–	–	–
1965	1	1	2	40	68	54

Aus den im Archiv überlieferten Aufnahmebüchern der Universitätsklinik Leipzig [40] geht hervor, dass im Jahr 1950 104 Patienten, im Jahr 1960 97 Patienten und im Jahr 1965 48 Patienten mit der Diagnose Schizophrenie aufgenommen wurden, wobei jedoch der Großteil der Akten dem Archiv nicht mehr zur Verfügung stand. Limitiert wurde die Anzahl der verfügbaren Akten, da ein großer Teil nicht auffindbar, zusätzlich durch Schimmelbildung entweder zerstört, in der Aufarbeitung war oder aufgrund des Zustandes nicht durch das Archiv freigegeben wurde. So gelang es leider nicht, für jeden Jahrgang eine gleiche oder ausreichende Anzahl an Akten zu gewinnen. Die Aktenführung selbst erschien kaum irgendeiner Standardisierung zu unterliegen. So waren zwar ein Aufnahmebefund und ein Entlassungsbrief vorhanden, weitere Einträge und deren Informationsgehalt unterlagen jedoch einer großen Bandbreite persönlicher Dokumentationsstile. Es wurde sich auf die Verwendung ärztlicher Informationen beschränkt. In einer Datenbank erfassten wir Basisdaten über den Patienten, dessen Aufenthalt, dessen psychopathologische Kriterien, den Entlassungs- oder Verlegungsort und die verwendeten Therapien. Oftmals erfolgte die Verabreichung von Antipsychotika ohne eine detaillierte Dokumentation.

## Eingesetzte Therapien an der Universität Leipzig 1946 bis 1965

**Figure 1:**
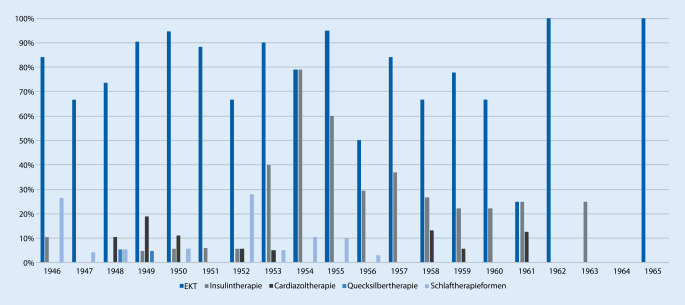

Die Schizophrenie- und Manietherapie an der Neurologisch-Psychiatrischen Universitätsklinik in Leipzig orientierte sich vor allem an den zeitgenössisch üblichen und sowohl in der BRD als auch in der sowjetischen Besatzungszone und der frühen DDR verbreitetsten Behandlungsformen. Eine gewisse Ausnahme bildet lediglich die Pawlow-Schlaftherapie, deren Protagonist allem voran Müller-Hegemann war. Über gute Erfolge dieser medikamentös induzierten Schlafverlängerung berichtet er vor allem bei Erschöpfungszuständen [27]. Wie sich nun zeigt, versuchte er jedoch mit seinem Amtsantritt als kommissarischer Direktor in Leipzig zum 01.06.1952 zunächst auch Erfahrungen mit dieser Therapieform bei schizophrenen und manischen Patienten zu sammeln, bevor sie 1963 wegen Todesfällen in der Klinik völlig eingestellt werden musste ([34]; Abb. 1).

**Figure 2:**
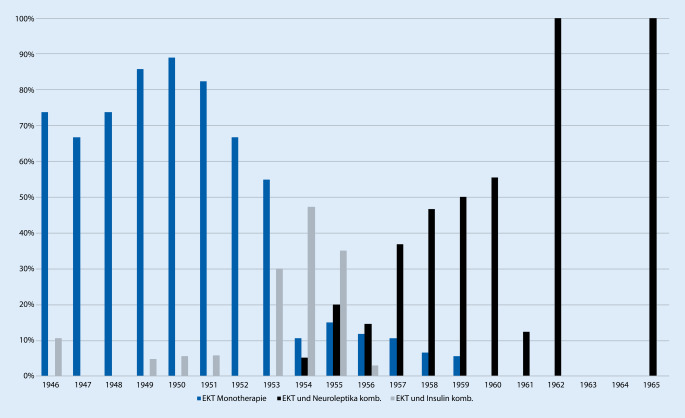

Allen voran wurde die seit 1938 eingeführte Elektrokonvulsionstherapie eingesetzt und gemäß der Lehrmeinung von Richard Arwed Pfeifer [30, 33], Amtsvorgänger Müller-Hegemanns [32], auch indikatorisch weit und kontinuierlich angewandt (Abb. 2). In den Jahren 1946 bis 1951 dominierte die Elektrokonvulsionstherapie deutlich und wurde in 65 bis 90 % der Fälle sogar als einzige Therapie eingesetzt. Die monotherapeutische Anwendung der EKT erreichte zwischen 1949 und 1951 ihren Höhepunkt und ging ab 1952 kontinuierlich zurück. Ab dem Jahr 1954 wurden nur noch um die 10 % der Patienten einzig mit EKT behandelt, ab dem Jahr 1960 keiner der untersuchten Fälle. Allerdings sind die Zahlen von 1962 und 1965 aufgrund der geringen in die Studie einbeziehbaren Fälle keineswegs repräsentativ. Der Verweis auf die Kombinationstherapie EKT und Antipsychotika als „Mittel der ersten Wahl“ bei auf Antipsychotika allein nicht ansprechenden Patienten fand in der Folge auch Eingang in Müller-Hegemanns Lehrbuch und scheint also der klinischen Erfahrung im Hause zu entsprechen.

Auch Medikamente, die „Heilkrämpfe“ auslösten, befanden sich für eine Doppeltherapie mit der Elektrokonvulsionstherapie im Behandlungsrepertoire, so Cardiazol, Anoixol oder das 1935 eingeführte Insulin. Es konnte ein zeitweiser Einsatz von Cardiazol zwischen 1948 und 1953 sowie 1958 bis 1961 in meist um die 10 %, einzig 1949 knapp 20 % der Fälle, stets in Kombination mit anderen Therapien, gefunden werden. Im Gegensatz zu Karl Leonhards an der Nervenklinik der Berliner Charité verfolgtem EKT-kritischem Therapieansatz einer Cardiazolkrampftherapie [24] entschieden sich die Leipziger Ärzte eher für den Einsatz des Insulins. Eine Insulinkrampftherapie als alleinige Form war jedoch nur in den Jahren 1952 bis 1954, 1956 und 1958 zu finden. Der Einsatz erfolgte in deutlich weniger als 10 % der Fälle. Ähnlich niedrige Behandlungsanwendungen erfuhr die Kombination aus Elektrokonvulsionstherapie und Insulinkrampftherapie in den Jahren 1946 bis 1952. Lediglich in den Jahren 1953 bis 1955 konnte eine deutliche Zunahme auf 30 bis sogar 47 % der Fälle (1954) gesehen werden, während danach die Einsatzhäufigkeit rasch wieder abnahm.

**Figure 3:**
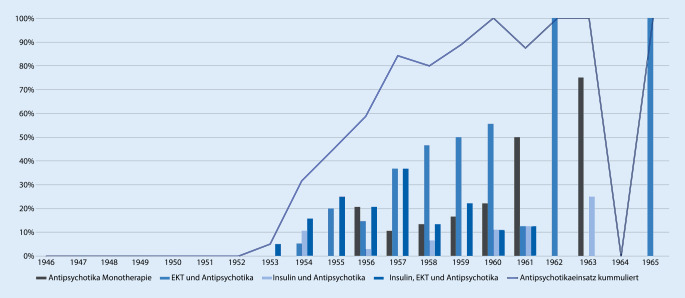

**Figure 4:**
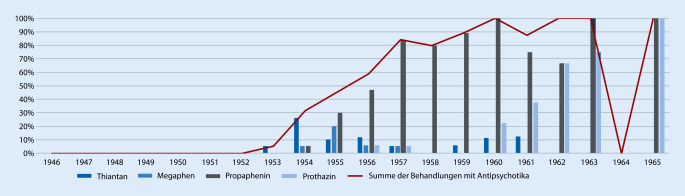

Die ersten Antipsychotika wurden in der untersuchten Klinik 1953 eingesetzt (Abb. 3). Gemäß Müller-Hegemanns Hinweis auf die Wirkung von Thiantan setzten die Leipziger Ärzte das Phenotiazin Thiantan in diesem Jahr erstmals bei der Behandlung der Schizophrenie ein (Abb. 4). In geringem Umfang konnte diese Medikation bis in das Jahr 1961 nachvollzogen werden. Chlorpromazin wurde erstmalig in Form des bundesdeutschen Megaphen 1954 bei 2 Fällen eingesetzt. Bereits im gleichen Jahr erfolgte der erste Einsatz des in der DDR hergestellten Propaphenin. Der Einsatz von Antipsychotika erfuhr einen steten Anstieg und erreichte erstmalig einen durchgehenden Einsatz ab 1960. Im Jahr 1961 erfolgte lediglich bei einer Patientin keine Behandlung, da sie auf Entlassung gedrängt hatte und rasch entlassen wurde. Der erste Einsatz des Antihistaminikums Prothazin mit D2-Rezeptor-Antagonismus erfolgte 1956 zusammen mit Propaphenin. Weitere Therapien, wie Quecksilbertherapie (2 Fälle) und Opiumkur (14 Fälle) spielten eine untergeordnete Rolle. Während anfangs der Einsatz von Chlorpromazin noch zusammen mit Elektrokonvulsionstherapie und Insulinkrampftherapie erfolgte, so nahm bis 1960 nicht nur die Einsatzhäufigkeit von Antipsychotika auf bis 100 % der Fälle zu, sondern vor allem auch ihre monotherapeutische Anwendung. Gleichzeitig fiel im gleichen Zeitraum bis 1960 die Anzahl der Fälle, bei denen eine Kombination aus Elektrokonvulsionstherapie und Insulinkrampftherapie angewandt wurde. Die 2er-Kombination von Elektrokonvulsionstherapie und Antipsychotikagabe konnte jedoch in steigender Häufigkeit festgestellt werden und fand durchgehend einen häufigeren Einsatz als eine ausschließliche Antipsychotikatherapie.

## Diskussion der Resultate

**Figure 5:**
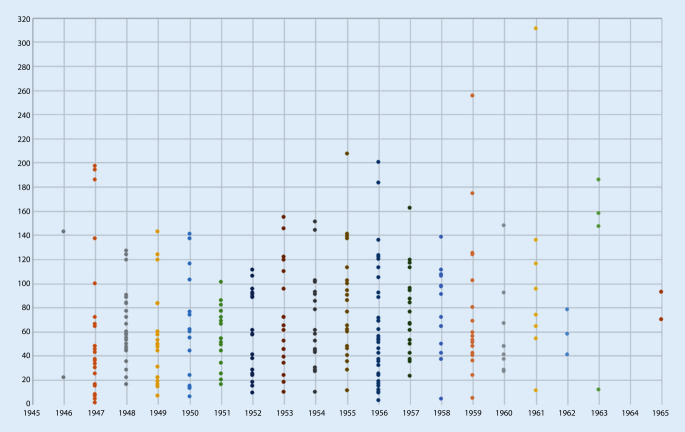

**Figure 6:**
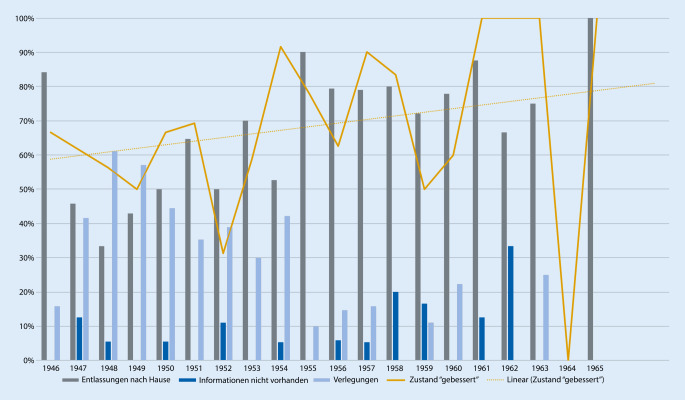

Mit Beginn des Bekanntwerdens einer neuen medikamentös wirksamen Behandlungsform scheint ab 1953 auch in Leipzig zügig ein praktisch-klinisch nachweisbarer Wandel hin zur antipsychotischen Therapie stattgefunden zu haben. Allerdings wurde sie erst 1956 erstmals als Monotherapie verabreicht und 1961 wurde dann erstmals auch nur die Hälfte der Fälle mit einer neuroleptischen Monotherapie behandelt. Die Antipsychotika wurden also zuerst mit vorhandenen Therapieformen kombiniert. So erfuhr vorrübergehend das Insulin wieder an Bedeutung. Gleichzeitig führte dies zu einer deutlichen Reduktion der EKT, die vordem die hauptsächliche Monotherapieform darstellte. Während bis 1952 sogar eine Kombination aus EKT und Insulin wenig gebräuchlich war, scheinen die Kombinationen sich ab 1953 deutlich zu diversifizieren und die Ärzteschaft legte eine große Probierfreudigkeit an den Tag. In den folgenden Jahren zeugen die Patientenakten von einem beständig neuen Kombinieren von einerseits antipsychotischer Medikation und andererseits einem traditionellen Behandlungsweg. Möglicherweise war der vorrübergehende Bedeutungsanstieg des Insulins ein Effekt der begrenzten Verfügbarkeit von Antipsychotika, welche sich bis in die 1960er-Jahre hineinzog [13]. Schließlich setzte sich die Kombination aus Antipsychotika und EKT als die am meisten verwendete Kombination durch, und die Behandlung mittels EKT und Insulin in Monotherapie oder Verbindung beider wurde in den Hintergrund verdrängt und verschwand ab 1960 völlig aus dem Therapierepertoire. Eventuell mag Müller-Hegemann auch eine etwas kritischere Ansicht zur Elektrokonvulsionstherapie als der um über 30 Jahre ältere Pfeifer gehabt haben. Gegen Letzteres spräche allerdings der Befund, dass die Kombination von EKT und Antipsychotikatherapie ab 1957 wieder zunimmt. Müller-Hegemann schilderte in seinem Lehrbuch von 1966 denn auch einen Einsatz der Phenothiazintherapie in Kombination mit einer Elektrokonvulsionstherapie als Mittel der ersten Wahl. Nur in den Fällen, wo eine solche Behandlung „nicht ans Ziel führt“, empfahl er eine Kombination mit einer Insulinkrampftherapie. In eben diesem Buch sprach Müller-Hegemann von einem „Siegeszug“ der Antipsychotika und einem Therapieerfolg, welcher sich „bedeutend vergrößert“ habe. Weiterhin beschrieb er, wenn auch mit kritischem Unterton, eine Verkürzung der Aufenthaltsdauer in der Psychiatrie durch den Einsatz von Antipsychotika [22]. Doch woran bemisst Müller-Hegemann diesen „Siegeszug“? Anhand einer reduzierten Verweildauer, einer Verlegung in Landesheilanstalten, einer Entlassung nach Hause oder subjektiv nach dem Zustand des Patienten bei seiner Entlassung? Werden die verbliebenen Patientenzahlen betrachtet, nachdem Verlegungen, Fluchten von Stationen und Todesfälle herausgenommen worden sind, ist an der Anzahl an Behandlungstagen zu erkennen, dass sich die Dauer einer Behandlung nicht deutlich in den Jahren von 1946 bis 1965 veränderte und die Einführung der Antipsychotikatherapie keine Auswirkung auf die Verweildauer der vorhandenen Fälle zu haben schien (*p* < 0,001; Abb. 5). Auch in den Jahren ab 1961 sind lange Behandlungszeiten festzustellen. Die Verlegungen chronischer, zur Heilung nicht Hoffnung gebender Patienten in die Landesheilanstalten bildeten lange den herkömmlichen Verfahrensweg der Universitätspsychiatrien, um Betten freizumachen – nicht zuletzt für forscherisch interessante Neuzugänge. Die Leipziger Universitätspsychiatrie war hier keine Ausnahme. Ab dem Jahr 1955 ist jedoch nun hier eine geringere Verlegungsrate in die Landesheilanstalten und eine erhöhte Entlassungsrate nach Hause zu beobachten (*p* < 0,01; Abb. 6). Somit kann in Leipzig nach Einführung der Antipsychotika von einem doch recht eindrücklichen Wandel hin zur vermehrten Entlassung zurück nach Hause ausgegangen werden. Gründe für zunehmende Entlassungen nach Hause und der Verzicht auf Verlegungen in die Landesheilanstalten können eine medikamentös geführte ambulante Versorgung oder auch eine erhöhte Wiederaufnahmebereitschaft der Patienten gewesen sein. Eine medikamentöse Therapie könnte im Vergleich zu den traditionellen Elektrokonvulsionstherapien und Insulinkrampfbehandlungen weniger abschreckend auf die Patienten gewirkt haben. Die Vermutung, dass die selteneren Verlegungen im Zusammenhang mit der zunehmenden Behandlung mittels Antipsychotika stehen, kann unterstützt werden durch den Fakt, dass 1955 tatsächlich der Einsatz von Antipsychotika erstmalig bei über der Hälfte der behandelten Patienten erfolgte. Aus den Akten geht andererseits kein klarer Anhaltspunkt für die Beweggründe des Verbleibs von Patienten in der Universitätspsychiatrie hervor. Auch erfolgte zum Beispiel die eine Verlegung im Jahr 1963 nach 12 Tagen ohne einen aus den Akten ersichtlichen Grund. Die Rate der festgestellten selteneren bzw. völlig ausbleibenden Verlegungen ab 1961 kann durch die geringe Anzahl an einbeziehbaren Patientenakten verzerrt sein. Für das Jahr 1956 standen mehr Patientenakten zur Verfügung als in den Jahren zuvor und danach, die gleichzeitig auch eine vollständige Behandlung in der Universitätspsychiatrie nachzeichneten. Ob dieser Verbleib auf ein gestiegenes Forschungsinteresse zurückzuführen war, kann angenommen, aber nicht belegt werden. Womöglich waren durch eine Besserung der Symptomatik die Patienten auch besser führbar und das Personal sah sich seltener veranlasst, schwere Fälle in Kliniken mit Pflegecharakter zu verlegen. Sedierende Medikamente wie Barbiturate wurden jedoch weiterhin unverändert eingesetzt (*p* 0,001). So fanden sich die Barbiturate Phenobarbital, Crotylbarbital, Hexobarbital und Cyclobarbital, jedoch auch Schlafmittel wie Chloralhydrat und Carbromal. In 5 Fällen fand sich das Stimulanz Pervitin, welches sich letztmalig 1958 nachweisen ließ.

Während anfänglich Phenothiazine in der Behandlung in Form einer zeitlich begrenzten „Kur“ mit bis zu 150 mg 3‑mal täglich eingesetzt wurden, so wandelte sich dies ab dem Jahr 1961. Es wurden nun vermehrt Patienten während der gesamten stationären Aufenthaltszeit mittels Antipsychotika in gleicher Dosierung behandelt. Durch die Möglichkeit, psychiatrische Medikamente auch außerhalb des Krankenhauses weiter zu verordnen, wurde eine Entlassung in die Gesellschaft unter fortlaufender Behandlung möglich, da mittels der neuen Medikamente psychotisch erkrankte Menschen nicht nur in einen symptomfreieren Zustand gebracht werden konnten, sondern es gab nunmehr ein Mittel, um die „gesunden Phasen“ ausdehnen zu können. Es fanden sich dokumentierte Entlassungsmedikationen mit einer Erhaltungsdosis von 3‑mal 75–100 mg Propaphenin am Tag. Die Angaben in den Akten über den Zustand der Patienten bei Entlassung sind kaum allgemein aussagekräftig auszuwerten. Oft fehlen diese Angaben gänzlich und wo sich Hinweise auffinden lassen, ist nicht klar, welche Kriterien zugrunde liegen. Es muss wohl von einer subjektiven Meinung des jeweiligen Arztes ausgegangen werden. Auch kann sich eine Verzerrung der Beurteilung über die Jahre ergeben, da sich die zu erreichende Qualität für „gebessert“ mit dem Wandel der Therapieoptionen grundlegend verändert haben könnte.

Auch wenn die traditionellen Behandlungsformen in den auf 1953 folgenden Jahren deutlich an Einsatzzahl sanken, kann resümiert werden, dass folgend ein Nebeneinander an Therapielinien bestand. Die Einführung von Antipsychotika bewirkte somit einen Wandel und eine Ergänzung in der psychiatrischen Behandlung in der Leipziger Neurologisch-Psychiatrischen Universitätsklinik, was zugleich zu einer Reduktion der Verlegungen in die Landesheilanstalten führte, ein weiteres Leben in der Mitte der Gesellschaft und Familie ermöglichte und zumindest die subjektive Entlassungsbeurteilung „nicht gebessert“ nicht nur reduzierte, sondern seit 1961 in den eingesehenen Akten sogar verhinderte (Abb. 6). Ein nicht näher bekannter Teil der Patienten möge noch eine ambulante Heilkrampftherapie erhalten haben. Die Akten der gesichteten Patienten enthielten jedenfalls keine solchen Einträge. Es ist nicht wahrscheinlich, aber möglich, dass hierfür dann separate Akten verwendet wurden. Müller-Hegemann schilderte in seinem Lehrbuch auch ambulante Heilkrampftherapien, bei welchen die Patienten lediglich für einige Stunden in der Ambulanz waren, um dort eine solche Therapie zu absolvieren.

## References

[1] Balz V, Osten P (2010). „Nervöse sind heilbar“. Die ersten Chlorpromazinversuche an der psychiatrischen Universitätsklinik Heidelberg im Jahr 1953 im Spiegel der Krankenakten und der Sicht von Arzt, Pflegepersonal und Patient. Patientendokumente. Krankheit in Selbstzeugnissen.

[2] Balz V (2010). Zwischen Wirkung und Erfahrung – eine Geschichte der Psychopharmaka. Neuroleptika in der Bundesrepublik Deutschland, 1950–1980.

[3] Balz V, Bürgi M, Eschenbruch N, Hulverscheidt M (2008). Magic bullets, chemical gagging, controlled risks? On the research of the network “Pharmaceuticals in the 20th century” of the German Research Foundation (DFG). Medizinhist J.

[4] Balz V, Hoheisel M (2011). East-side story: the standardisation of psychotropic drugs at the Charité Psychiatric Clinic, 1955–1970. Stud Hist Philos Biol Biomed Sci.

[5] Balz V, Klöppel U (2015). Wendung nach Innen. Vierteljahrsh Zeitgesch.

[6] Bangen HC (1992). Geschichte der medikamentösen Therapie der Schizophrenie.

[7] Delay J, Deniker P, Ropert R (1956). Study of 300 case histories of psychotic patients treated with chlorpromazine in closed wards since 1952. Encephale.

[8] Ewald G (1948). Lehrbuch der Neurologie und Psychiatrie.

[9] Grage H (1955). Klinische Erfahrungen mit Megaphen (= Propaphenin) unter besonderer Berücksichtigung als Antiepilepticum und als Hilfsmittel bei Vergiftungen. Psychiatr Neurol Med Psychol.

[10] Heidrich R (1952). Zur Klinik des protrahierten Insulinkomas. Psychiatr Neurol Med Psychol.

[11] Helmchen H (1990). Psychiatrische Diagnostik ex juvantibus?. Nervenarzt.

[12] Hess V (2007). Psychochemicals crossing the wall. Die Einführung der Psychopharmaka in der DDR aus der Perspektive der neueren Arzneimittelgeschichte. Medizinhist J.

[13] Klöppel U, Eschenbruch N, Balz V, Klöppel U, Hulverscheidt M (2010). Brigade Propaphenin arbeitet an der Ablösung des Megaphen. Der prekäre Beginn der Psychopharmaka-Produktion in der DDR. Arzneimittel des 20. Jahrhunderts.

[14] Klöppel U, Balz V (2010). Psychopharmaka im Sozialismus. Arzneimittelregulierung in der Deutschen Demokratischen Republik in den 1960er Jahren. Ber Wissenschaftsgesch.

[15] Lautenschläger CL (1955). 50 Jahre Arzneimittelforschung.

[16] Lemke R, Rennert H (1965). Neurologie und Psychiatrie.

[17] Meduna L (1938). Vierjährige Erfahrungen mit der Cardiazol-Konvulsionstherapie.

[18] Meyer HH (1953). Die Winterschlafbehandlungin der Psychiatrie und Neurologie. Dtsch Med Wochenschr.

[19] Mosig A (1955). Importierte Arzneimittel. Pharm Prax.

[20] Müller M, Bally G (1963). Die Insulinbehandlung. Grundlagen und Methoden der Klinischen Psychiatrie.

[21] Müller-Hegemann D (1954). Bericht über die Arbeitstagung der Staatlichen Pawlow-Kommission der DDR vom 15.–17.11.1954 in Leipzig. Diskussionsbemerkung zu Thiantan und Megaphen. Psychiatr Neurol Med Psychol.

[22] Müller-Hegemann D (1966). Neurologie und Psychiatrie. Lehrbuch für Studierende und Ärzte.

[23] Rempen E, Linde OK (1988). Megaphen – die Einführung des ersten Neurolpetikums in der Bundesrepublik Deutschland. Pharmakopsychiatrie im Wandel der Zeit.

[24] Rzesnitzek L (2015). „Schocktherapien“ und Psychochirurgie in der frühen DDR. Nervenarzt.

[25] Sakel M, Pötzl O (1935). Neue Behandlungsmethoden der Schizophrenie.

[26] Salm H (1949). Untersuchungen zur Frage der Gedächtnis- und Merkfähigkeitsstörungen nach Elektrokrampfbehandlung. Psychiatr Neurol Med Psychol.

[27] Scholtz D, Steinberg H (2011). Die Theorie und Praxis der Pawlow’schen Schlaftherapie in der DDR. Psychiatr Prax.

[28] Schulte W (1949). Die Elektroschockbehandlung. Psychiatr Neurol Med Psychol.

[29] Sigwald J, Bouttier D (1953). Le chlorhydrate de chloro-3 (dimethylamino-3’-propyl)-10-phénothiazine en pratique neuro-psychiatrique courante. Bull Mem Soc Med Hop Paris.

[30] Somburg O, Steinberg H (2008). Richard Arwed Pfeifer. Die Ästhetik schizophrener Kunst und die Hirnforschung. Nervenarzt.

[31] Steinberg H (2016). 25 Jahre nach der „Wiedervereinigung“: Versuch einer Übersicht über die Psychiatrie in der DDR. Teil 1: Nachkriegszeit, Pawlowisierung, psychopharmakologische Ära und sozialpsychiatrische Bewegung. Fortschr Neurol Psychiatr.

[32] Steinberg H (2018). Die Karriere des Psychiaters Dietfried Müller-Hegemann (1910–1989). Beispiel eines politisch gewollten Auf- und Abstiegs in der DDR. Nervenarzt.

[33] Steinberg H, Carius D, Himmerich H (2013). Richard Arwed Pfeifer—a pioneer of “medical pedagogy” and an opponent of Paul Schroder. Hist Psychiatry.

[34] Steinberg H, Weber MM (2011). Vermischung von Politik und Wissenschaft in der DDR. Die Untersuchung der Todesfälle an der Leipziger Neurologisch-Psychiatrischen Universitätsklinik unter Müller-Hegemann 1963. Fortschr Neurol Psychiatr.

[35] Vogt W (1956). Beitrag zum klinischen Vergleich der Wirksamkeit von Largactil und Serpasil. Schweiz Arch Neurol Psychiatr.

[36] Walther R (1949). Elektroschock und vegetatives Nervensystem. Psychiatr Neurol Med Psychol.

[37] Weber MM (1999). Die Entwicklung der Psychopharmakologie im Zeitalter der naturwissenschaftlichen Medizin.

[38] Whitaker-Azmitia P (1999). The discovery of serotonin and its role in neuroscience. Neuropsychopharmacology.

[39] Universitätsarchiv Leipzig, Bestand Patientenakten der Neurologisch-Psychiatrischen Universitätsklinik Leipzig. Jahre 1946–1965.

[40] Universitätsarchiv Leipzig, Bestand Patienten-Aufnahmebücher der Neurologisch-Psychiatrischen Universitätsklinik Leipzig. Jahre 1946–1965.

