# Erratum zu: Die Einführung der Antipsychotika an der Neurologisch-Psychiatrischen Klinik der Universität Leipzig und ihre Auswirkungen auf andere Therapieformen sowie auf die Verweildauern und Verlegungen

**DOI:** 10.1007/s00115-021-01236-4

**Published:** 2021-12-03

**Authors:** Christian Oeser, Holger Steinberg

**Affiliations:** 1 Forschungsstelle für die Geschichte der Psychiatrie, Klinik und Poliklinik für Psychiatrie und Psychotherapie, Medizinische Fakultät der Universität Leipzig, Semmelweisstr. 10, 04103 Leipzig, Deutschland; 2 Klinik für Psychiatrie und Psychotherapie im Fachkrankenhaus Hubertusburg, Wermsdorf, Deutschland

**Erratum zu:**

**Der Nervenarzt 2020**

10.1007/s00115-020-00931-y

Der Artikel „Die Einführung der Antipsychotika an der Neurologisch-Psychiatrischen Klinik der Universität Leipzig und ihre Auswirkungen auf andere Therapieformen sowie auf die Verweildauern und Verlegungen“ von Christian Oeser und Holger Steinberg wurde ursprünglich Online First ohne „Open Access“ auf der Internetplattform des Verlags publiziert. Nach der Veröffentlichung in Band 92 Heft 1 pp. 69–80 hatten sich die Autoren für eine „Open-Access“-Veröffentlichung entschieden. Das Urheberrecht des Artikels wurde deshalb in © Der/die Autor(en) 2020 geändert. Dieser Artikel ist jetzt unter der Creative Commons Namensnennung 4.0 International Lizenz veröffentlicht, welche die Nutzung, Vervielfältigung, Bearbeitung, Verbreitung und Wiedergabe in jeglichem Medium und Format erlaubt, sofern Sie den/die ursprünglichen Autor(en) und die Quelle ordnungsgemäß nennen, ein Link zur Creative Commons Lizenz beigefügen und angeben, ob Änderungen vorgenommen wurden.

Die in diesem Artikel enthaltenen Bilder und sonstiges Drittmaterial unterliegen ebenfalls der genannten Creative Commons Lizenz, sofern sich aus der Abbildungslegende nichts anderes ergibt. Sofern das betreffende Material nicht unter der genannten Creative Commons Lizenz steht und die betreffende Handlung nicht nach gesetzlichen Vorschriften erlaubt ist, ist für die oben aufgeführten Weiterverwendungen des Materials die Einwilligung des jeweiligen Rechteinhabers einzuholen.

Weitere Details zur Lizenz entnehmen Sie bitte der Lizenzinformation auf http://creativecommons.org/licenses/by/4.0/deed.de.

